# Editorial Comment to “Geriatric Nutritional Risk Index Predicts Postoperative Complications in Elderly Patients Undergoing Robot‐Assisted Radical Cystectomy”

**DOI:** 10.1111/iju.70123

**Published:** 2025-05-23

**Authors:** Kenji Zennami, Shusuke Akamatsu

**Affiliations:** ^1^ Department of Urology Nagoya University Graduate School of Medicine Nagoya Japan

Open radical cystectomy (RC) is one of the most invasive surgeries in the urological field, with high blood loss and complication rates. To reduce the invasiveness, especially in elderly patients, robot‐assisted RC (RARC) has been widely applied and is steadily increasing due to the refinement of surgical procedures and the aging population. In addition, intracorporeal urinary diversion (ICUD) and enhanced recovery after surgery (ERAS) have gained popularity because they have also been shown to reduce gastrointestinal complications, including ileus, blood loss, and length of stay [1]. However, in real‐world practice, the rate of complications, particularly in the elderly remains relatively high; therefore, the establishment of a predicting system for perioperative complications is an imperative matter.

In the present study, Fukuta K et al. retrospectively evaluated the effectiveness of preoperative Geriatric Nutritional Risk Index (GNRI) as a marker for predicting 90‐day postoperative complications in elderly patients who underwent RARC [2]. Patients with an abnormal GNRI (< 92) had a significantly higher rate of 90‐day postoperative complications (*p* < 0.001) and an abnormal GNRI was a significant predictor of 90‐day postoperative complications (odds ratio: 9.963; 95% confidence interval: 2.125–46.718; *p* = 0.004). These findings showed the suitability of preoperative GNRI assessment for RARC in elderly patients.

The geriatric assessment (GA) consists of various assessment items, such as functional ability, nutrition, comorbidities, cognitive ability, psychosocial disorders, polypharmacy, social and financial support, falls/imbalance, and vision/hearing, with each distinct measure [3]. GNRI is one of the GA tools which focuses more on nutritional status. Besides GNRI, Geriatric‐8 (G8) is a famous, useful GA tool widely used in the urological field [3]. Previous studies reported that postoperative ileus (POI) still exists at a substantial rate even after RARC with ICUD (iRARC) and frail patients with a low G8 score had a significantly higher risk of POI [4]. Moreover, the ERAS protocol did not reduce POI in frail patients after iRARC, although it enhanced bowel recovery and reduced POI in non‐frail patients [5]. These findings indicate that frailty is a robust, unmodifiable risk factor for postoperative complications, and the improvement of surgical procedures and perioperative management protocols has a limit to reducing those complications. Refinement of risk predicting tools (e.g., GNRI, G8) as well as the establishment of a frailty‐based management strategy including prehabilitation, antibiotic prophylaxis, and specific surgical procedures are indispensable for better management of patients undergoing RARC, particularly in highly aging countries.

## Author Contributions

**Kenji Zennami**: writing – original draft, review, and editing. **Shusuke Akamatsu**: editing and supervision.

## Conflicts of Interest

The authors declare no conflicts of interest.
